# Nosip is a potential therapeutic target in hepatocellular carcinoma cells

**DOI:** 10.1016/j.isci.2023.107353

**Published:** 2023-07-10

**Authors:** Junjie Gao, Dandan Yang, Zheng Huang, Xueshan Pan, Ruoxue Cao, Chaoqun Lian, Jia Ma, Yuyun Li, Zhiwei Wang, Jun Xia

**Affiliations:** 1 Bengbu Medical College Key Laboratory of Cancer Research and Clinical Laboratory Diagnosis, School of Laboratory Medicine, Bengbu Medical College, Bengbu 233030, Anhui, China; 2 Department of Clinical Laboratory, the Second Affiliated Hospital of Wannan Medical College, Wuhu 241001, Anhui, China; 3 Anhui Province Key Laboratory of Immunology in Chronic Diseases, Bengbu Medical College, Bengbu 233030, Anhui, China; 4 Department of Biochemistry and Molecular Biology, School of Laboratory Medicine, Bengbu Medical College, Bengbu 233030, Anhui, China; 5 Department of Laboratory, Lianyungang Second People’s Hospital, Lianyungang 222000, Jiangsu, China; 6 Department of Clinical Laboratory Diagnostics, School of Laboratory Medicine, Bengbu Medical College, Bengbu, Anhui 233030, China

**Keywords:** Therapeutics, Cancer

## Abstract

Nitric oxide synthase-interacting protein (Nosip) interacts with nitric oxide synthase (NOS) and regulates NO synthesis and release, which participates in various critical physiological and pathological processes. However, the role of Nosip in hepatocellular carcinoma (HCC) is unclear. In this study, Nosip expression was found to be elevated in HCC tissues and cells. Nosip siRNA transfection inhibited the proliferation and motility of HCC cells and promoted apoptosis. In contrast, overexpression of Nosip promoted proliferation and migration and invasion, and inhibited apoptosis of HCC cells. As a natural compound, quercetin exerted the effect of inhibiting the proliferation and motility of HCC cells, and this anticancer activity probably via repressing the expression of Nosip. Our results suggest that Nosip could act as an oncogene in the progression of HCC and that quercetin may be a potential natural compound for treating HCC by inhibiting the expression of Nosip.

## Highlights

• Nosip participates in various critical physiological and pathological processes
• Nosip expression is elevated in HCC tissues and cells
• Overexpression of Nosip promotes proliferation and motility of HCC cells
• Quercetin exerts the anticancer activity via inhibition of Nosip in HCC

## Graphical abstract

**Figure:**
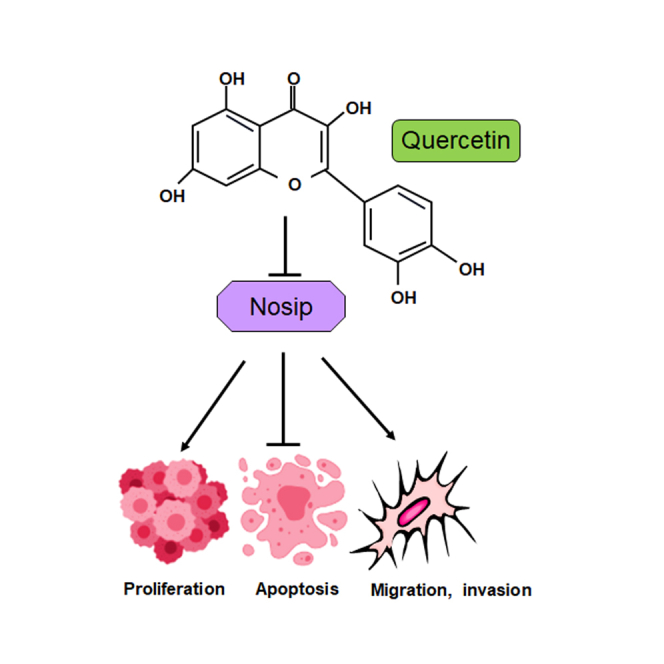

## Introduction

Hepatic cell carcinoma (HCC) is the common malignant tumor in the world and the second deadliest malignant tumor worldwide.^1^ Epidemiological investigations have revealed that most HCC is caused by chronic hepatitis B or hepatitis C virus.^2^ Because HCC is highly malignant with a poor prognosis, radiotherapy and chemotherapy are the common treatments.^3^ Sorafenib, a multikinase inhibitor, as a first-line drug in the treatment of advanced liver cancer has prolonged the survival time to some extent; however, its serious side effects and drug resistance still need to be eliminated.^4^ Surgical treatment is the mainstay for early-to-intermediate stage liver cancer patients, but five years survival rate after surgical resection is only 10%.^5^ Therefore, we urgently need to find the potential diagnostic markers of HCC for the early diagnosis and explore effective drugs for the treatment of HCC.

Nitric oxide synthase-interacting protein (Nosip), also known as CGI-25, can interact with nitric oxide synthase (NOS) and widely exists in eukaryotic cells.^6^^,^^7^^,^^8^^,^^9^ Nosip can regulate the activity and localization of NOS,^6^ promote the transfer of endothelial nitric oxide synthase (eNOS) from the cell membrane to the cytoplasm, uncouple eNOS from sites on the plasma membrane, and thus inhibit the synthesis of nitric oxide (NO).^10^ Nosip can regulate the homeostasis of NO in physiological and pathological pain.^11^ Studies have shown that Nosip and eNOS can be distributed on non-vascular cells in the stomach and intestines to regulate gastrointestinal secretion and movement.^12^ Nosip expression is increased in patients with congenital colon and involved in enterocolitis development by suppressing local NO production in patients with Hirschsprung’s disease.^13^ Nosip is ubiquitously expressed in the tracheal epithelium and lung tissue and plays an essential role in regulating mucociliary and bronchial function by controlling NO synthesis in airways and blood vessels.^14^ When the expression of Nosip is decreased, the production of NO will be interfered, and the abnormal concentration of NO will cause vascular remodeling and pulmonary hypertension.^15^ In neuroepithelioma cell lines, Nosip reduces neuronal nitric oxide synthase activity, leading to inhibition of NO production.^7^ Nosip is a crucial factor in the developing and self-renewal of neural stem cells or neural crest cells in Xenopus and mice.^16^^,^^17^ It plays an essential role in astrocyte formation after spinal cord injury.^9^ Nosip has been reported to act as a ubiquitin ligase that mediates the ubiquitination of PP2A catalytic subunits, and knockout of the Nosip gene causes increased activity of PP2A in mouse craniofacial tissues, resulting in holoprosencephaly and facial dysmorphism.^18^ Different doses of NO release may affect tumor development^19^^,^^20^^,^^21^^,^^22^; the latest study also suggested that Nosip may affect granulocytic differentiation and thereby influence the progression of chronic myelogenous leukemia.^23^ As a regulator of NO synthesis and release, whether Nosip can play a role in developing HCC is worth investigating.

The natural compound quercetin is a kind of flavonoid widely found in fruit and vegetable.^24^ Quercetin has been widely studied in recent years because of its promising anti-inflammatory,^25^ antioxidant,^26^ as well as anticancer effects.^27^ Researchers have found that quercetin can exert its corresponding tumor suppressor effects in prostate, breast, ovarian, colorectal, lung, and esophageal cancers through regulating multiple pathways.^28^^,^^29^^,^^30^^,^^31^^,^^32^^,^^33^ However, there are few studies regarding the role and molecular mechanism of quercetin in HCC. The purpose of this study is to explore the functions of Nosip in HCC and to test whether quercetin as a natural compound can treat HCC via Nosip pathway. Specifically, we first studied the expression of Nosip in HCC tissue by bioinformatics, and then clarified that Nosip as a cancer-promoting factor may play an essential role in the occurrence and development of HCC. Finally, we evaluated the inhibitory effect of quercetin on the malignant biological function of HCC. We found that anticancer activity of quercetin may be achieved by regulating the expression of Nosip.

## Results

### Nosip expression in patients with HCC

**Figure 1:**
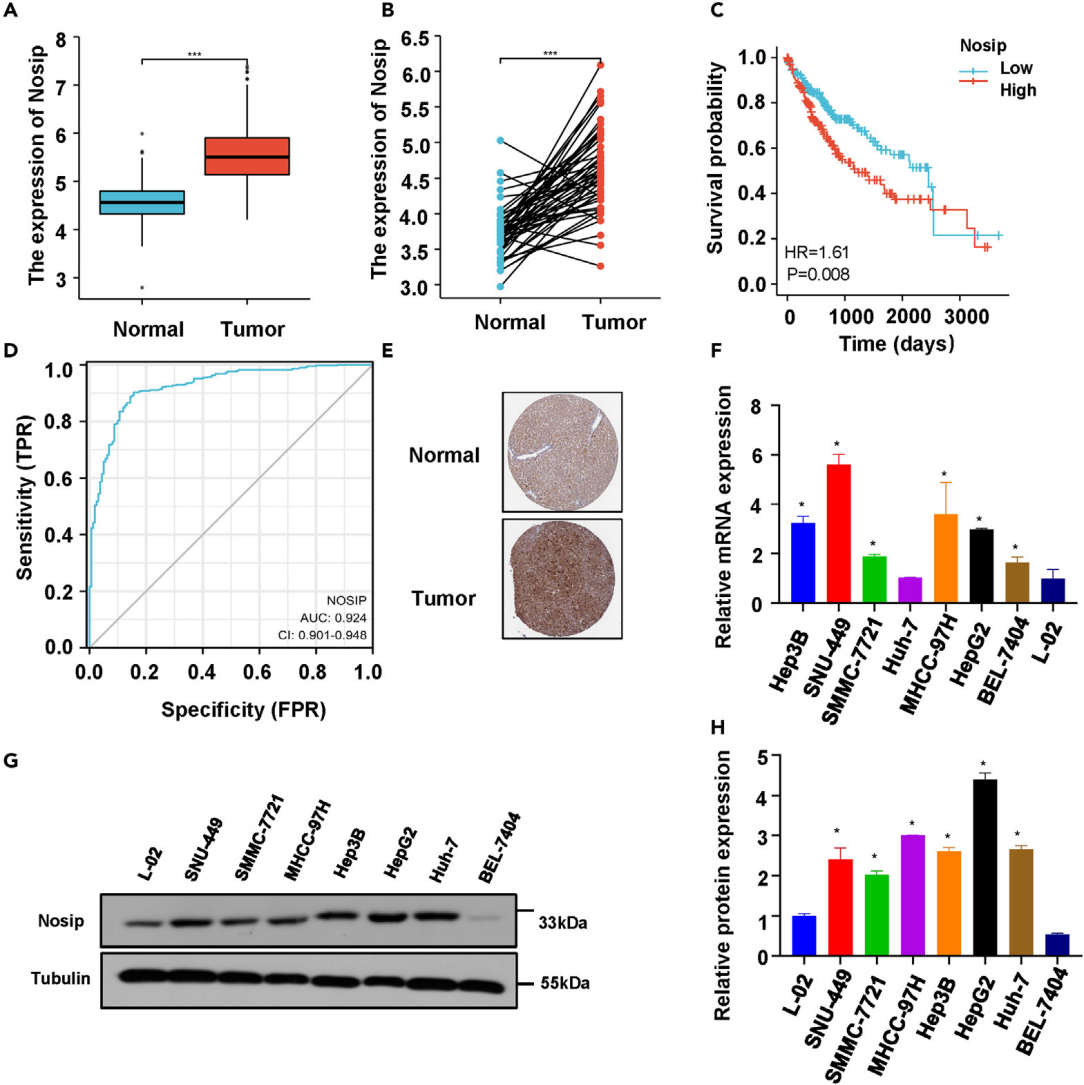
Nosip expression in HCC (A) Nosip mRNA expression in HCC samples and normal tissues are illustrated (Tumor = 371, Normal = 160, p < 0.001). (B) Nosip mRNA expression in HCC and paired adjacent normal tissues are illustrated (n = 50, p < 0.001). (C) Kaplan-Meier survival analysis was conducted between patients with high and low expression levels of Nosip (n = 373, p = 0.008). (D) Receiver operating characteristic analysis (ROC) of Nosip in patients with HCC is illustrated (AUC = 0.924). (E) The level of Nosip protein in HCC tissue by immunohistochemistry analysis was lower than that in normal tissue in the Human Protein Atlas. (F) Real-time PCR was performed to measure the Nosip mRNA levels in seven different HCC cells and normal liver cell L-02. (G) Western blot analysis was performed to measure the protein levels of Nosip in seven different HCC cells and normal liver cell L-02. (H) The quantitative data for the protein levels of Nosip in panel G.

To determine the role of Nosip in HCC progression, the mRNA expression data and clinical follow-up data of 371 patients with HCC and 160 normal liver tissues were downloaded from TCGA and GTEX databases to compare with the expression of Nosip in tumor group and non-tumor group. The results showed that Nosip was significantly upregulated in tumor group (Figure 1A), and the same results were obtained in tumor group and its paired adjacent tissues (Figure 1B). Kaplan-Meier analysis showed that higher expression of Nosip was significantly associated with shorter overall survival in patients with HCC (Figure 1C). The expression ROC curve showed that the expression level of Nosip had a specific diagnostic value in the occurrence of HCC (Figure 1D). Immunohistochemistry data showed that the staining intensity of Nosip in HCC tissues was higher than that in normal liver tissue (Figure 1E). In order to study the biological function of Nosip in HCC, we carried out cell experiments *in vitro*. First of all, we detected the expression level of Nosip in 7 different HCC cell lines and normal hepatocytes by real-time qPCR and western blotting. The results showed that the expression of Nosip was increased in most HCC cells, which was consistent with the expression level of Nosip in HCC tissues (Figures 1F–1H). Among them, a higher level of Nosip was expressed in Hep3B and SNU-449 cells, while a lower level of Nosip was expressed in SMCC-7721 cells (Figures 1F–1H).

### Nosip effect on proliferation and apoptosis of HCC cells

**Figure 2:**
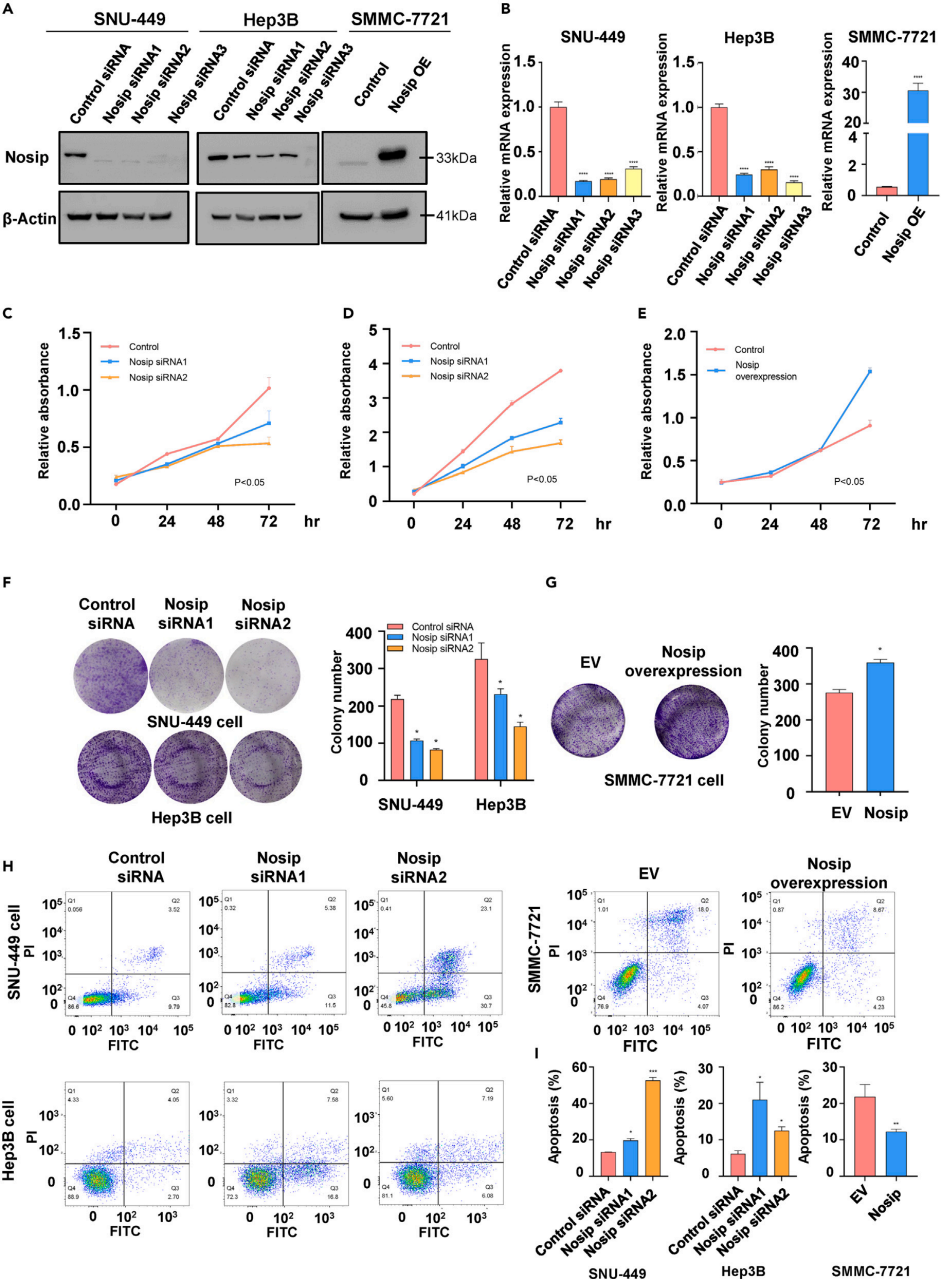
Nosip effects on proliferation and apoptosis of HCC cells (A) The efficacy of Nosip siRNA or Nosip cDNA transfections on the expression of Nosip in HCC cells was analyzed by western blotting analysis. (B) The efficacy of Nosip siRNA or Nosip cDNA transfections on the mRNA level of Nosip in HCC cells was analyzed by RT-qPCR. p < 0.0001. (C) Cell viability assay (CCK-8 assay) was performed in SNU-449 cells transfected with control siRNA and Nosip siRNAs for 24, 48, and 72 h. p < 0.05. (D) CCK-8 assay was performed in Hep3B cells transfected with control siRNA and Nosip siRNAs for 24, 48, and 72 h. p < 0.05. (E) Cell viability assay (CCK-8 assay) was performed in SMCC-7721 cells transfected with vector plasmids and Nosip overexpression plasmids for 24, 48, and 72 h. p < 0.05. (F) Left panel: Colony-forming assay was performed in SNU-449 cells and Hep3B cells after Nosip siRNA transfection. Right panel: The quantitative results for colony formation are illustrated. p < 0.05. (G) Left panel: Colony-forming assay was performed in SMCC-7721 cells after Nosip cDNA transfection. Right panel: The quantitative results for left panel are illustrated. p < 0.05. (H) Apoptosis was measured in SNU-449 cells and Hep3B cells after Nosip siRNA transfection, and SMCC-7721 cells after Nosip cDNA transfection. (I) The quantitative results for apoptosis (H) are illustrated. ∗p < 0.05, ∗∗p < 0.01, ∗∗∗p < 0.001.

To investigate whether Nosip affects cell growth, we transfected Nosip siRNAs into Hep3B and SNU-449 cells. SMCC-7721 was transfected with Nosip-overexpressing (OE) plasmid. Western blotting and RT-qPCR results showed that Nosip expression was decreased in Hep3B and SNU-449 cells after siRNA transfection, while Nosip expression was increased in SMCC-7721 cells after Nosip OE plasmid transfection (Figures 2A and 2B). CCK-8 assay showed that downregulation of Nosip significantly inhibited the proliferation of Hep3B and SNU-449 cells (Figures 2C and 2D), while overexpression of Nosip enhanced the proliferation ability of SMCC-7721 cells (Figure 2E). In addition, clone formation experiments showed that the downregulation of the Nosip could significantly inhibit the clone formation of Hep3B and SNU-449 cells (Figure 2F). Compared with empty vector-treated SMCC-7721 cells, clone formation capacity of the Nosip OE group was significantly increased (Figure 2G). Consistent with these results, downregulation of Nosip promoted the apoptosis of HCC cells (Figure 2H), whereas overexpression of Nosip inhibited the apoptosis of HCC cells (Figure 2I). These results suggest that Nosip can promote cell proliferation and inhibit cell apoptosis in HCC.

### Nosip effect on HCC cell migration and invasion

**Figure 3:**
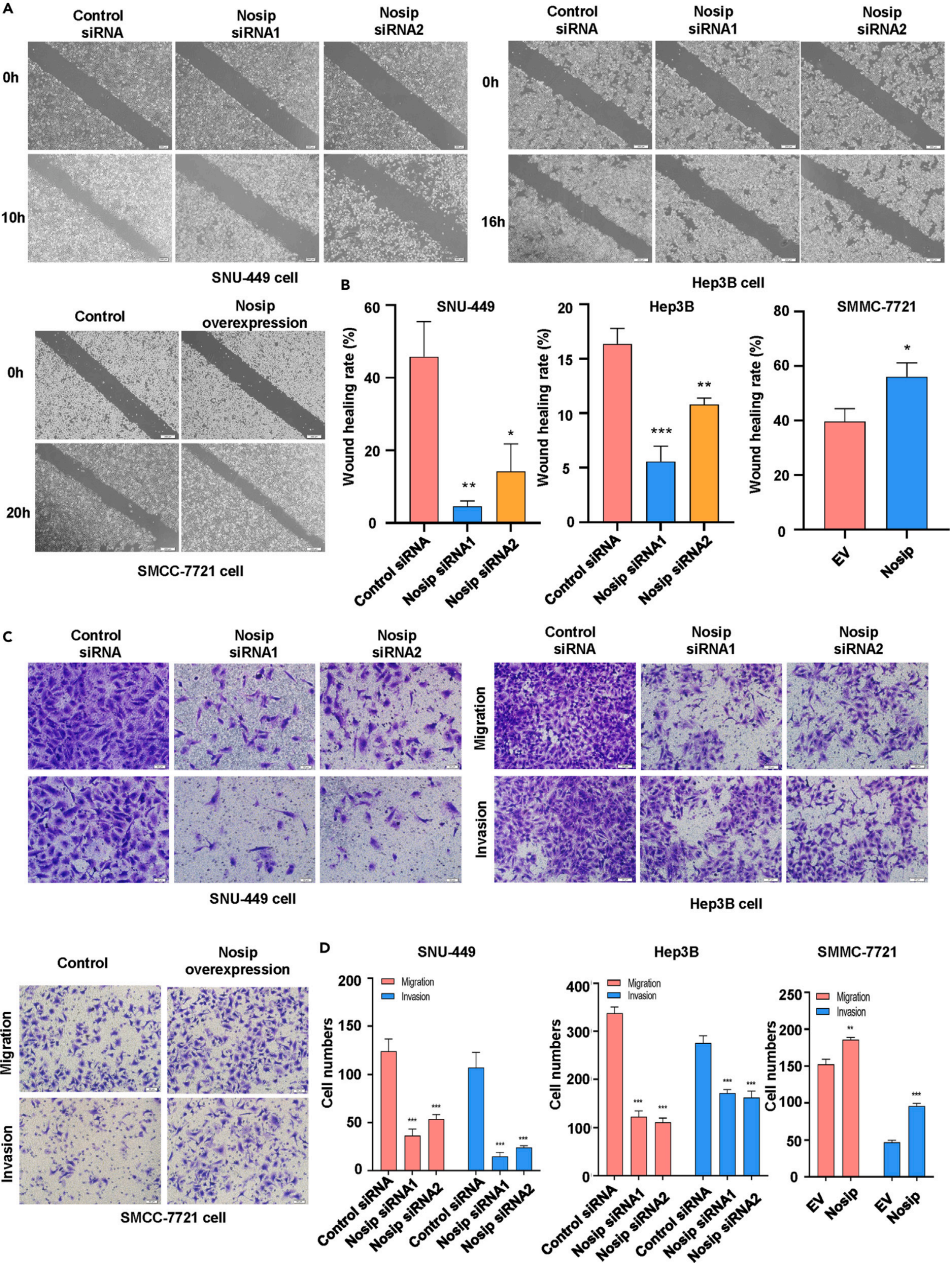
Nosip effects on the migration and invasion of HCC cells (A) Wound healing assay was performed to measure the migratory capacity in SNU-449 cells and Hep3B cells after Nosip siRNA transfection and in SMCC-7721 cells after Nosip cDNA transfection for 10, 16, and 20 h, respectively. (B) Quantitative data are illustrated for wound healing assay on panel A. (C) Transwell migration and invasion assays were performed to measure the migratory and invasive capacity in SNU-449 cells and Hep3B cells after Nosip siRNA transfection and in SMCC-7721 cells after Nosip cDNA transfection for 24 h. (D) Quantitative data are illustrated for migration and invasion results on panel C. ∗p < 0.05, ∗∗p < 0.01, ∗∗∗p < 0.001).

To further investigate the effects of Nosip on the motility, migration, and invasion abilities of HCC, we examined the effects on wound healing of HCC cells after Nosip was downregulated or overexpressed. The results showed that the wound healing abilities of HCC cells were significantly weakened when Nosip was downregulated (Figure 3A), whereas the wound healing abilities were greatly strengthened after overexpression of Nosip (Figure 3B) To verify the role of Nosip in HCC cell migration and invasion, Transwell chamber assays were performed to detect cell migration and invasion. Our results showed that Nosip downregulation inhibited HCC cell migration and invasion (Figures 3C and 3D), whereas Nosip overexpression promoted this process (Figures 3C and 3D). These data suggest that Nosip can regulate the motility of HCC cells.

### Quercetin effect on cell proliferation and apoptosis

**Figure 4:**
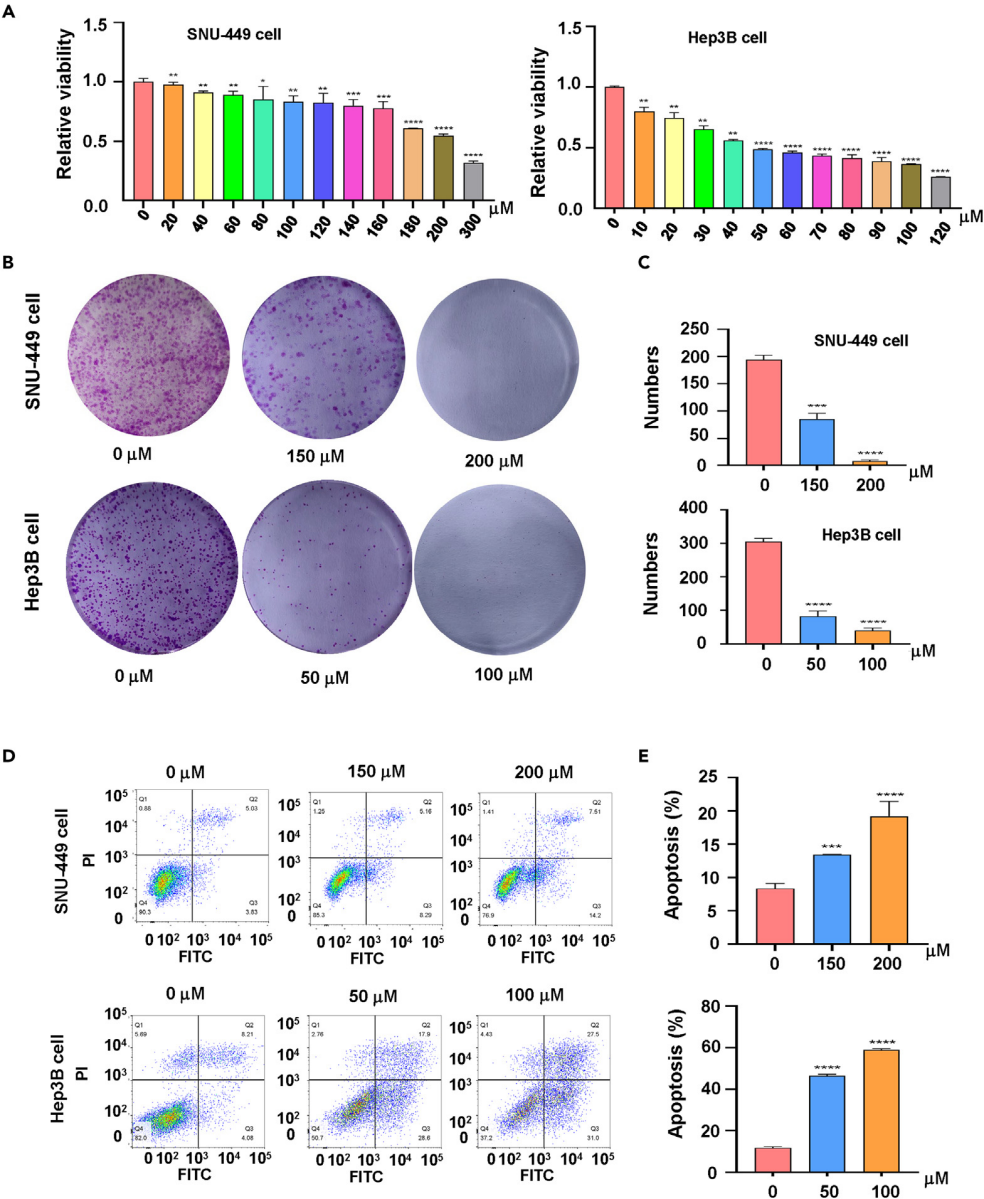
Quercetin effects on proliferation and apoptosis of HCC cells (A) Effects of quercetin with different concentrations on the viability of SNU-449 cells and Hep3B cells for 48 h were measured using the CCK-8 assay. (B) Colony formation test was carried out in SNU-449 cells and Hep3B cells treated with quercetin at different concentrations (50, 100, 150, 200 μM). (C) Quantitative data are illustrated for colony formation on panel B. (D) Cell apoptotic death in SNU-449 cells and Hep3B cells with quercetin treatment (50, 100, 150, 200 μM) at 48 h was tested by the Annexin V-FITC/PI approach. (E) Quantitative data are illustrated for cell apoptosis on panel D. ∗p < 0.05, ∗∗p < 0.01, ∗∗∗p < 0.001, ∗∗∗∗p < 0.0001 vs. the control.

To investigate whether quercetin has an inhibitory effect on the proliferation of HCC cells, we examined the cell viability after different doses of quercetin acted on SNU-449 and Hep3B for 48 h using the CCK-8 assay. We found that the effect of quercetin on the viability of HCC cells was concentration dependent (Figure 4A). Quercetin did not show the same sensitivity on the two cell lines; specifically, quercetin inhibited SNU-449 cells by about 20% at 150 μM, while it inhibited Hep3B cells by up to 80% at 50 μΜ. Quercetin at 50 μM reached 50% inhibition on Hep3B cells without significant inhibition on SNU-449 cells. To more comprehensively investigate the effects of different concentrations of quercetin on HCC cells, in the subsequent experiments, we selected 150 and 200 μM quercetin to treat SNU-449 cells. The inhibitory rates of quercetin on hepatoma cell SNU-449 at these two concentrations were about 20% and 45%, respectively, which were less than 50%. Hep3B cells were selected to be treated with 50 and 100 μM quercetin, and the inhibitory effects of quercetin on Hep3B at these two concentrations with about 50% and 70% inhibition. To clarify the effect of quercetin on the proliferation of HCC cells, we performed a clone formation assay. We found that the clone formation ability was significantly weakened with the increase of quercetin concentration, and the cell colony formation ability was inhibited considerably (Figures 4B and 4C). Then, to clarify the effect of quercetin on the apoptosis of HCC cells, we detected the apoptosis of SNU-449 and Hep3B cells at 48 h after the impact of different concentrations of quercetin. Annexin V-FITC/PI data showed that quercetin induced the apoptosis of SNU-449 and Hep3B cells (Figures 4D and 4E). In conclusion, quercetin can inhibit the cell proliferation and induce apoptosis of HCC cells.

### Quercetin effect on migration and invasion of HCC cells

**Figure 5:**
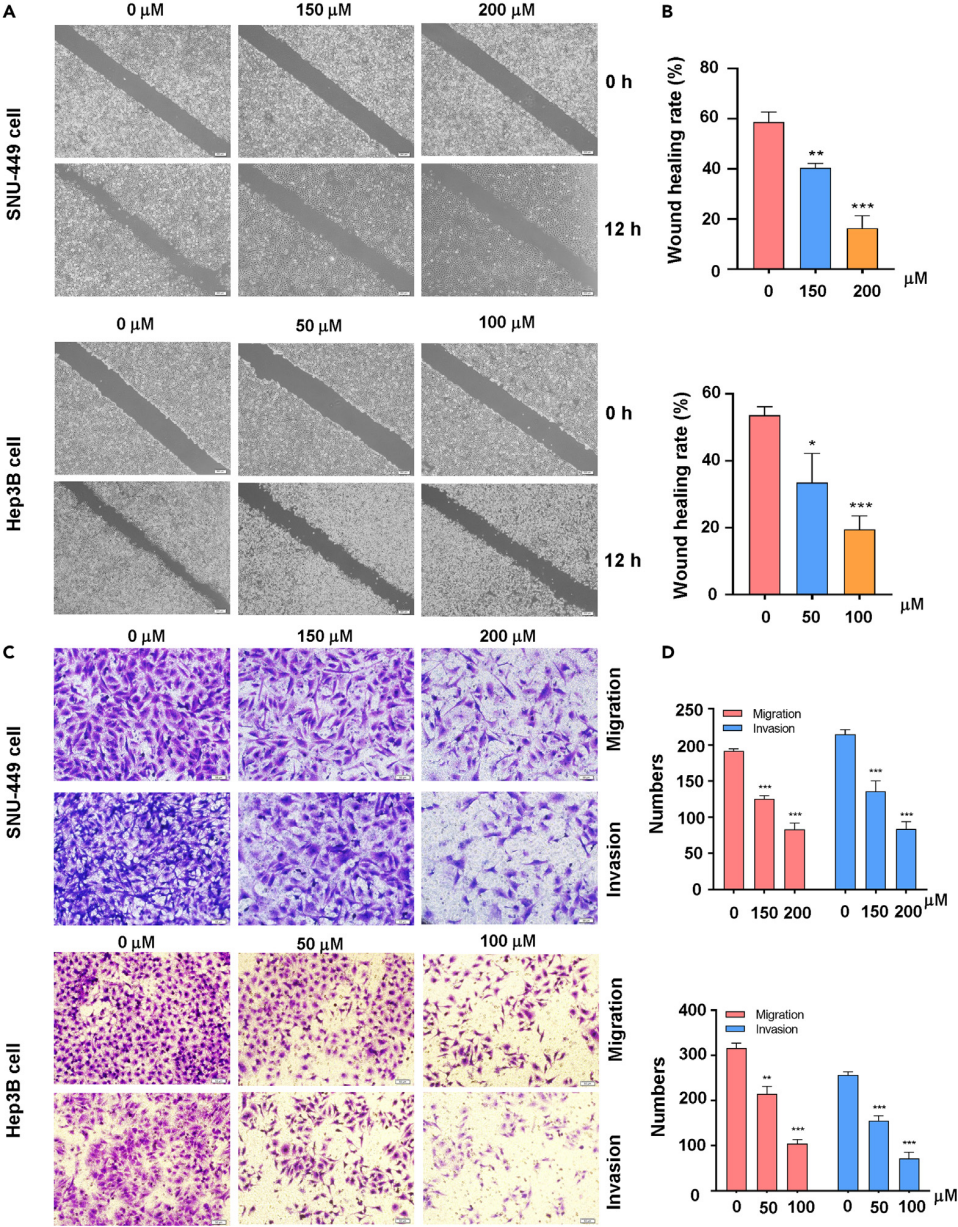
Quercetin effects on the migration and invasion of HCC cells (A) The wound healing test was used to measure the motility of SNU-449 cells and Hep3B cells treated with different concentrations of quercetin (50, 100, 150, 200 μM) for 12 h. (B) Quantitative data are illustrated for wound healing results on panel A. (C) Transwell migration and invasion assays were performed to measure the migratory and invasive capacity in SNU-449 cells and Hep3B cells after treatment with different concentrations of quercetin (50, 100, 150, 200 μM) for 24 h. (D) Quantitative data are illustrated for migration and invasion results on panel C. ∗p < 0.05, ∗∗p < 0.01, ∗∗∗p < 0.001 vs. the control.

Next, we examined whether quercetin inhibits the movement, migration, and invasion of HCC cells. Wound healing experiments showed that quercetin exposure reduced the wound healing ability of SNU-449 and Hep3B cells (Figures 5A and 5B). Transwell chamber migration and invasion experiments showed that quercetin exposure weakened the migration and invasion ability of SNU-449 and Hep3B cells (Figures 5C and 5D). The results showed that quercetin treatment decreased the motility, migration, and invasion of HCC cells.

### Quercetin effect on the expression of Nosip in HCC cells

**Figure 6:**
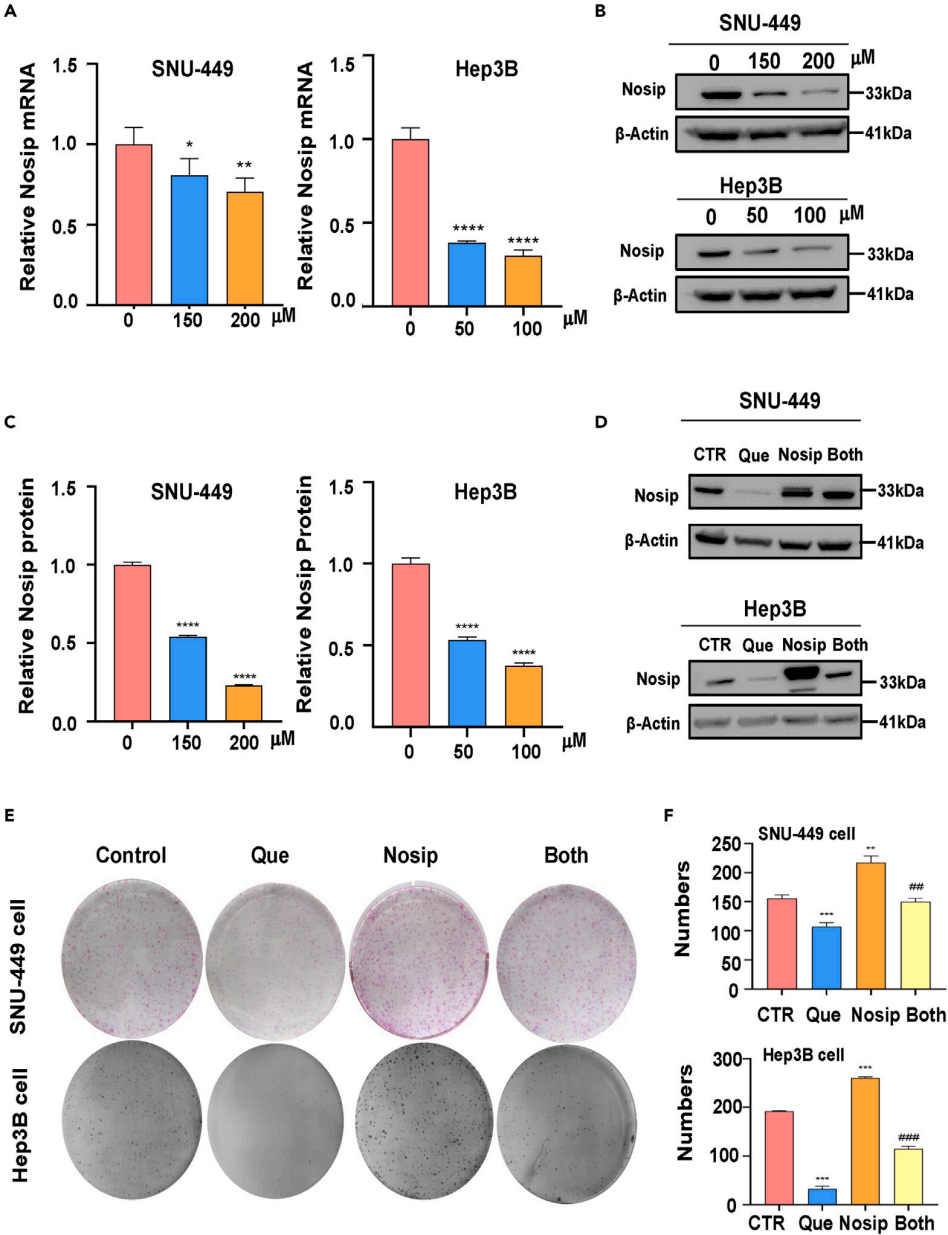
Quercetin effects on Nosip expression in HCC cells (A) The Nosip mRNA levels of SNU-449 cells and Hep3B cells treated with different concentrations of quercetin (50, 100, 150, 200 μM) for 48 h were detected by RT-qPCR. (B) The Nosip protein levels of SNU-449 cells and Hep3B cells treated with different concentrations of quercetin (50, 100, 150, 200 μM) for 48 h was detected by western blot analysis. (C) Quantitative data were illustrated for Nosip protein levels on panel B. (D) Nosip protein levels were detected by Western blot analysis in SNU-449 cells and Hep3B cells treated with quercetin (150 μM for SNU-449 cells, 50 μM for Hep3B cells) or Nosip cDNA plasmid or combination for 48 h. (E) Colony formation test was carried out in SNU-449 cells and Hep3B cells treated with quercetin (150 μM for SNU-449 cells, 50 μM for Hep3B cells) or Nosip cDNA plasmid or combination. (F) Quantitative data are illustrated for colony formation results on panel E. CTR: control group; Que: quercetin alone treatment group; Nosip: Nosip cDNA transfection group; Both: Nosip cDNA transfection plus quercetin treatment group. ∗p < 0.05, ∗∗p < 0.01, ∗∗∗∗p < 0.0001 vs. the control. ^##^p < 0.01, ^###^p < 0.001 vs. quercetin only or Nosip plasmid transfection only.

Next, we tested whether Nosip could be a critical carcinogenic protein in the occurrence and development of HCC. We investigated whether quercetin inhibited the malignant biological function of HCC cells by downregulating the expression of Nosip in HCC cells. The expression of Nosip in SNU-449 and Hep3B cells after quercetin treatment was analyzed by RT-qPCR and western blotting. Our data showed that quercetin inhibited the expression of Nosip in SNU-449 and Hep3B cells at mRNA (Figure 6A) and protein levels (Figures 6B and 6C). This finding suggests that the inhibition of Nosip by quercetin may be one of the reasons by which quercetin inhibits the malignant biological function of HCC cells.

### The effect of Nosip overexpression on quercetin function

**Figure 7:**
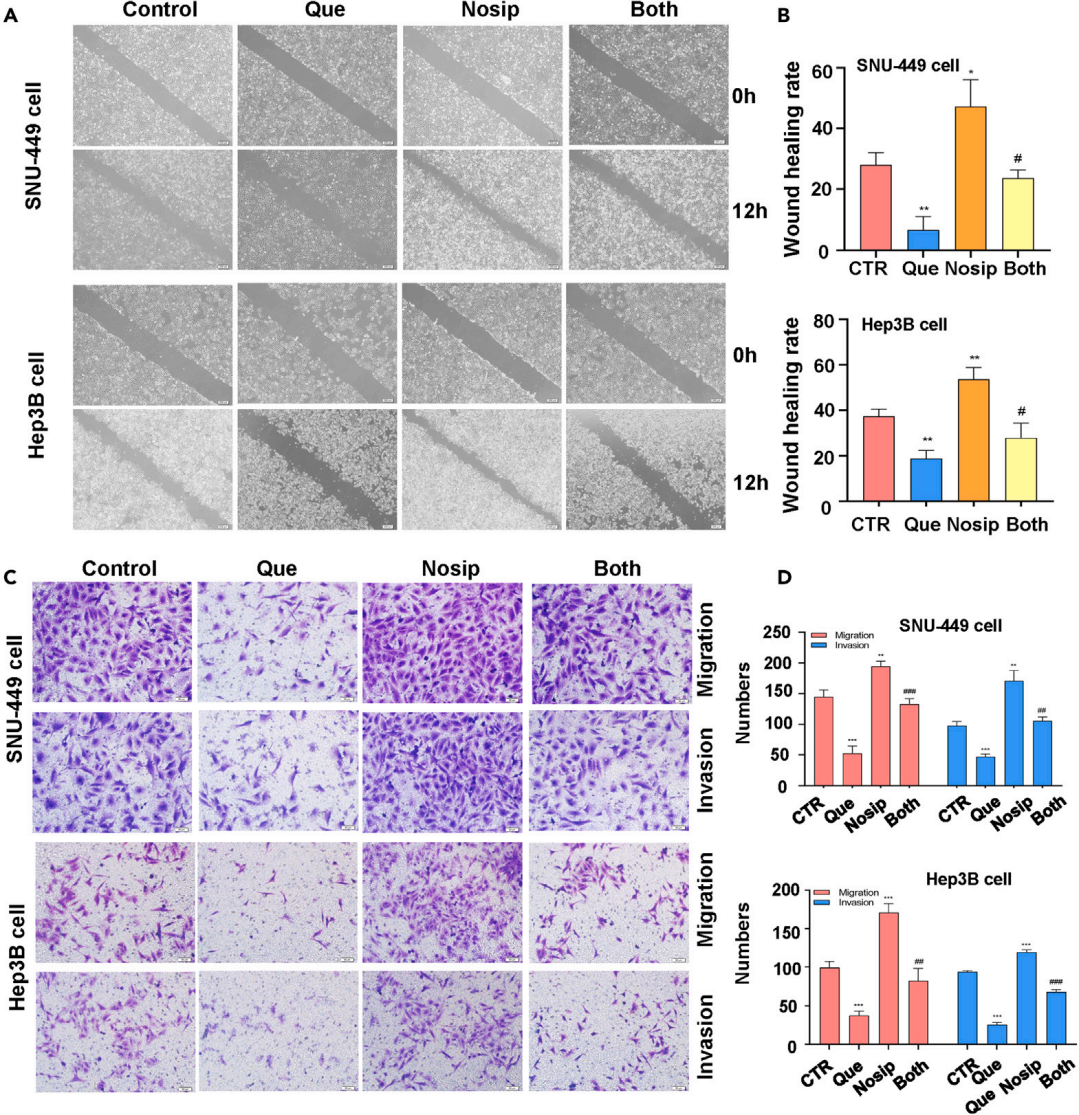
The effects of Nosip overexpression on quercetin function (A) The wound healing test was used to measure the motility of SNU-449 cells and Hep3B cells treated with quercetin (150 μM for SNU-449 cells, 50 μM for Hep3B cells) or Nosip cDNA plasmid or combination for 24 h. (B) Quantitative data are illustrated for wound healing results on panel A. (C) Transwell migration and invasion assays were performed to measure the migratory and invasive capacity in SNU-449 cells and Hep3B cells after treatment with quercetin (150 μM for SNU-449 cells, 50 μM for Hep3B cells) or Nosip cDNA plasmid or combination for 24 h. (D) Quantitative data are illustrated for migration and invasion results on panel C. ∗∗p < 0.01, ∗∗∗p < 0.001 vs. the control. ^#^p < 0.05, ^##^p < 0.01, ^###^p < 0.001 vs. quercetin only or Nosip plasmid transfection only.

In order to study whether the antitumor effect of quercetin is mediated by regulating Nosip, we transfected Nosip cDNA plasmid into HCC cells to elevate the expression level of Nosip. We tested whether the upregulation of Nosip can reverse the antitumor effect of quercetin on HCC cells. Western blotting showed that Nosip cDNA plasmid-transfected SNU-449 and Hep3B cells increased the expression of Nosip in SNU-449 and Hep3B cells (Figure 6D). Nosip cDNA transfection saved the reduction of Nosip in SNU-449 and Hep3B cells caused by quercetin treatment (Figure 6D). Next, we evaluated whether the increase of Nosip abolished quercetin-triggered inhibition of HCC cell activity. Clone formation data showed that Nosip overexpression promoted the colony formation of SNU-449 and Hep3B cells (Figures 6E and 6F), while Nosip overexpression neutralized the decline of HCC cell colony formation caused by quercetin exposure (Figure 6E). Wound healing assays showed that Nosip overexpression promoted the wound healing capacity of SNU-449 and Hep3B cells, and consistent upregulation of Nosip abolished the quercetin exposure-induced attenuation of the wound healing capacity (Figures 7A and 7B). Transwell was used to observe the migration and invasion of HCC cells with Nosip cDNA simultaneously after quercetin exposure. Nosip upregulation promoted the migration and invasion abilities of SNU-449 and Hep3B cells, whereas Nosip overexpression reversed the attenuation of HCC cell migration and invasion abilities caused by quercetin exposure (Figures 7C and 7D). Taking the previous experimental results together, we conclude that the antitumor effect of quercetin was partially achieved by attenuating the expression of Nosip in HCC.

### The effect of Nosip on NO accumulation in HCC

**Figure 8:**
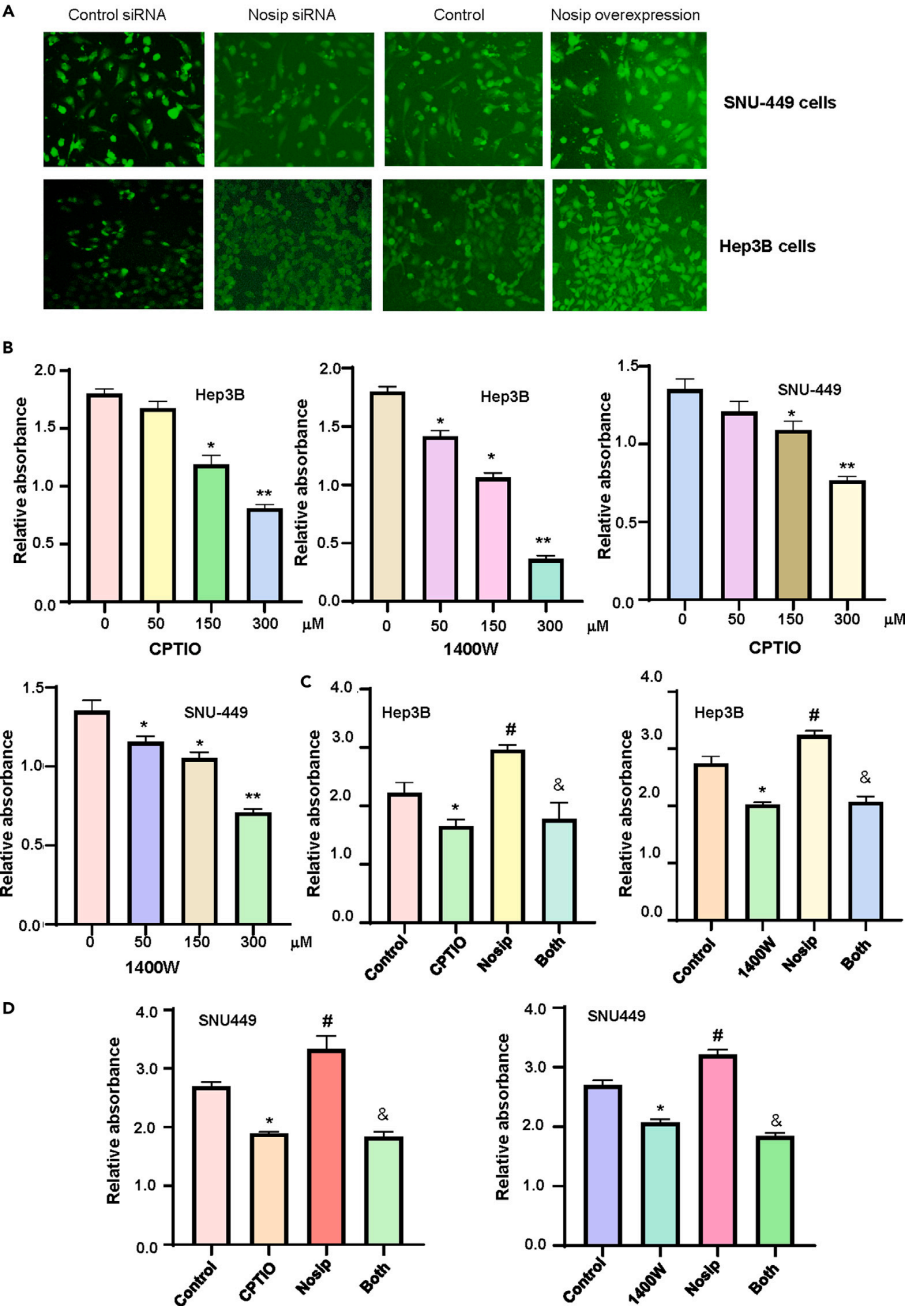
The effect of Nosip on NO accumulation in HCC (A) NO levels were measured by DAF-2DA in SNU-449 cells and Hep3B cells after Nosip siRNA transfection and Nosip cDNA transfection for 48 h, respectively. (B) CCK-8 assay was performed to measure cell viability in Hep3B cells and SNU-449 cells after CPTIO and 1400W treatments with different concentrations (50, 150, 300 μM) for 72 h. (C) CCK-8 assay was used to measure cell viability in Hep3B cells after treatment with CPTIO (150 μM), 1400W (150 μM), Nosip cDNA plasmid or combination for 72 h. (D) CCK-8 assay was used to measure cell viability in SNU-449 cells after treatment with CPTIO (150 μM), 1400W (150 μM), Nosip cDNA plasmid or combination for 72 h.

The effect of Nosip modulation on level of intracellular NO in HCC cells was detected using DAF-2DA. We found that overexpression of Nosip in SNU-449 and Hep3B cells induced intracellular NO levels (Figure 8A). Consistently, knockdown of Nosip in SNU-449 and Hep3B cells decreased intracellular NO levels (Figure 8A). To explore whether NO was involved in Nosip-mediated oncogenesis in HCC cells, we performed the CCK-8 assay in Hep3B and SNU-449 cells after treatments with CPTIO, 1400W, and Nosip cDNA plasmid. We observed that CPTIO, an NO scavenger, inhibited viability of Hep3B and SNU-449 cells (Figure 8B). Moreover, 1400W, an iNOS activity inhibitor, was found to reduce cell viability in both Hep3B and SNU-449 cells (Figure 8B). Furthermore, overexpression of Nosip increased viability of Hep3B and SNU-449 cells, which was abrogated by CPTIO and 1400W treatments (Figures 8C and 8D). Further investigation is necessary to dissect the association of Nosip, NO, iNOS, and eNOS in HCC cells.

## Discussion

In the present study, we reported that Nosip plays an oncogenic role in HCC, and quercetin could inhibit the HCC malignant biological function by inhibiting the expression of Nosip. Quercetin, as a natural flavonoid, has attracted much attention for its potent anti-inflammatory and anticancer effects.^34^ We utilized *in vitro* cell experiments to confirm that quercetin could inhibit the proliferation and motility and promote the apoptosis of HCC cells. We found that quercetin caused a decrease in the expression of Nosip after acting on HCC cells.

Nosip was discovered 20 years ago by researchers through a yeast two-hybrid system, which functions as a regulator to inhibit NO synthesis.^6^ Recent studies have also found that NO as a small molecule signaling molecule can participate in tumorigenesis.^35^ However, numerous studies have shown that NO exerts both pro- and tumor-suppressive effects that seem to be related to the concentration and time of action: high concentrations of NO cause DNA damage and mitochondrial dysfunction,^36^ while low concentrations of NO have the effects of stimulating angiogenesis and resisting apoptosis to promote tumorigenesis.^37^ Researchers found that 150 nM can be used as a threshold concentration to define whether NO exerts pro- or tumor-suppressive effects, specifically when the concentration of NO is below 150 nM, NO will activate protein kinase G (PKG), protein kinase B. The activated PKG can, in turn, promote the expression of metalloproteinase-9.^38^ At the same time, high NO can act with hypoxia-inducible factor 1α to increase vascular endothelial growth factor production,^39^ thereby promoting angiogenesis and endothelial cell proliferation and producing an anti-apoptotic response.^40^^,^^41^ While the tumor suppressor p53 is phosphorylated and activated when NO is higher than 150 nm, mitogen-activated protein kinase 1 is overexpressed, and cellular respiration is inhibited, thereby causing tumor cell death.^40^^,^^42^^,^^43^^,^^44^ Our experimental results confirmed that Nosip is highly expressed in HCC cancer cells. Knockdown of Nosip expression in HCC cells inhibited proliferation and motility and induced apoptosis, whereas overexpression of Nosip in HCC cells led to the opposed phenotype. Nosip modulation altered NO accumulation in HCC cells. CPTIO and 1400W treatments abolished Nosip overexpression-induced cell viability. We proposed that overexpression of Nosip could regulate NO production and promote tumorigenesis and development, which should be further validated by cell experiments and mouse models in the future.

Quercetin, one of six subclasses of flavonoids, is widely found in fruits and vegetables like apples and onions.^45^ Because quercetin has antiviral and anti-inflammatory effects, its role in liver diseases and cardiovascular diseases has been intensively studied in recent years.^46^^,^^47^ Studies have shown that quercetin can induce apoptosis in HepG2 cells^48^ and inhibit the proliferation, metastasis, and promote apoptosis and affect autophagy in HCC LM3 cells by regulating the JAK2/STAT3 signaling pathway.^49^ Quercetin nanoparticles can also inactivate Akt and ERK1/2 signaling pathways.^50^ Wnt/β-Catenin, PI3K/Akt, and NF-κB signaling pathways are also inhibited by quercetin, resulting in inhibiting hepatocarcinogenesis.^51^^,^^52^^,^^53^ It is known that the anti-hepatoma effects of quercetin are through targeting multi-pathways and multi-targets, while the specific molecular mechanisms need to be further studied. Our results showed that quercetin exerted antitumor activity by suppression of Nosip expression in HCC.

### Limitations of the study

In conclusion, our study shows that Nosip can promote the proliferation, migration, and invasion of HCC. Quercetin could be a useful agent to treat HCC via targeting Nosip. Nosip could be regarded as a biomarker to guide the early diagnosis and treatment of patients with HCC. In this study, we dissected the role of Nosip in HCC for the first time, which was approved by *in vitro* cell experiments that Nosip may function as a pro-oncogene in HCC. Although we uncovered that quercetin could inhibit Nosip expression in HCC cells and lead to anticancer function *in vitro*, the mouse model should be used to validate the role of Nosip and function of quercetin *in vivo*. The molecular mechanism of Nosip-mediated oncogenesis in HCC needs further in-depth exploration. These studies will help to find biomarkers for the early diagnosis of HCC and provide molecular mechanism support for the future clinical application of quercetin.

## STAR★Methods

### Key resources table

**Table:** 

REAGENT or RESOURCE	SOURCE	IDENTIFIER
**Antibodies**		

Anti-NOSIP	Proteintech	Cat# 27979-1-AP; RRID: AB_2881027
Anti-Tubulin	CST	Cat#5335;
HRP- conjugated β-actin	Proteintech	Cat#HRP-66009; RRID:AB_2883836

**Chemicals, peptides, and recombinant proteins**		

RPMI-1640 medium	Gibco	Cat# 11875119
McCoys 5A medium	Gibco	Cat# 16600082
DMEM medium	Gibco	Cat# 11965092
Quercetin	Sigma	Cat# PHR1488

**Experimental models: Cell Lines**		

Hep3B	Shanghai Cell Bank	Cat# SCSP-5045
L-02	Shanghai Cell Bank	Cat# BFN608006124
SNU-449	Shanghai Cell Bank	Cat# BFN60800681
SMCC-7721	Shanghai Cell Bank	Cat# BFN60800687

**Critical commercial assays**		

NOSIP siRNA	Gene Pharma	NA
PrimeScript cDNA synthesis kit	vazyme	Cat# R323-01
QIAquick PCR purification Kit	vazyme	Cat# Q711-02
NOSIP plasmid	Youbio	NA
Lipofectamine 3000	ThermoFisher	Cat# L3000008
Lipofectamine 2000	ThermoFisher	Cat# 11668019
DAF-2 DA	上海翌圣	CAS NO.:205391-02-2
1400W Dihydrochloride	MCE	Cat# HY-18731; Lot No.28897
Carboxy-PTIO potassium	MCE	Cat#HY-18734; Lot No.157072

**Software and algorithms**		

GraphPad Prism	GraphPad	Version 8.0
Flowjo	BD Biosciences	Version 10
Image J	NIH	https://imagej.nih.gov/ij/

**Others**		

Transcriptomic data of HCC	TCGA	https://portal.gdc.cancer.gov/

### Resource availability

#### Lead contact

Further information and requests for resources should be directed to the lead contract, Jun Xia (xiajun@bbmc.edu.cn).

#### Materials availability

This study did not generate new unique reagents.

### Experimental model and subject details

#### Cell lines and cell culture

Hepatoma cell lines Hep3B, Huh-7, HepG2, SNU-449, SMCC-7721, MHCC-97h, BEL-7404, and liver normal epithelial cells L-02 were purchased from Shanghai Cell Bank, Chinese Academy of Sciences. Cells were maintained in culture medium with 10% fetal bovine serum (Clark) and placed in an incubator with 5% carbon dioxide at 37°C. Both DMEM and RPMI-1640 medium were purchased from Gibco Company. CCK-8 kits were purchased from Beyotime Company. Quercetin reagent was obtained from Sigma Company. The anti-Nosip antibody was obtained from Proteintech Company, while anti-β-actin antibody was purchased from Abclonal Company.

#### Information for clinical samples

The mRNA expression data of human HCC were downloaded from TCGA database (https://portal.gdc.cancer.gov/), while mRNA expression data of normal liver tissues were downloaded from the GTEX database (www.gtexportal.org/). A total of 371 cases of HCC tissue samples and 160 cases of liver normal tissue samples were obtained after the data were merged and homogenized. From the human protein atlas website (https://www.proteinatlas.org), HCC tissues and normal liver tissue immunohistochemistry results were downloaded.

### Method details

#### Expression difference analysis

The mRNA data downloaded from databases were subjected to name conversion and log2 correction to obtain the expression of Nosip in tumor group and adjacent nontumor group. The expression of the two groups was statistically analyzed using R software ggplot2 package, limma package, and beeswarm package. The box diagram and pairing diagram were made to visualize the analysis results.

#### Survival analysis

Patient survival status, survival time, and Nosip gene expression were extracted from HCC clinical prognostic information. Patients were categorized into two groups with high and low expression according to the median mRNA expression. Survival analysis and visualization of patients were performed using the R software survival package and the survminer package.

#### Transfection

The two siRNAs for Nosip were transfected into two hepatoma cells, SNU-449 and Hep3B cells, using the transfection reagent Lipofectamine 2000 (Genepharma, Shanghai, China). Cells were divided into three groups: NC, Nosip siRNA1, Nosip siRNA2 groups. The plasmid for Nosip (Genepharma, Shanghai, China) was transfected into SMCC-7721 cells using Lipofectamine 3000.^54^ The siRNA sequences targeting Nosip were as follows: siRNA1 5‘-CCU ACC ACG AGA AGA AGA ATT-3’, 5‘-UUC UUC UUC UCG UGG UAG GTT-3’; siRNA2 5‘-CCC AGA UGA UGU CCA ACC UTT-3’, 5‘-AGG UUG GAC AUC AUC UGG GTT-3’.

#### CCK-8 analysis

Hepatoma cells were cultured in 6-well plates for 24 h and then transfected or treated with drugs. After continuous culture for 24 h, 48 h, and 72 h, 5000 treated cells were transferred to 96-well plates. 10 μl of CCK-8 reagent was added to each well at 24 h, 48 h, and 72 h, and then placed in a 37°C incubator for further incubation for 3 h. Finally, the absorbance of cells in 96-well plates at 450 nm was measured by a microplate reader (BioRad Laboratories Inc, CA, USA) as described previously.^55^

#### Clone formation experiment

HCC cells were transfected with plasmids or treated with drugs for different times. Cells were digested with pancreatin, and then 1000 HCC cells were removed and placed in a new 6-well plate and continued to culture for 7∼10 days until apparent colony formation was observed. After the medium was discarded, cells were washed several times with PBS, and fixed using 4% paraformaldehyde, stained using 0.1% crystal violet for 10 min at room temperature. The colonies were photographed after washed and air dried.

#### Apoptosis analysis by flow cytometry

5 x 10^5^ transfected or treated HCC cells were plated into 60 mm dishes for 48 h or 72 h. Cells were digested using 0.25% pancreatin and washed by cold PBS for third times. Cells were transferred to flow tubes, stained using Annexin V-FITC/propidium iodide for 15 min in dark at room temperature. Apoptosis was assessed using flow cytometry (BD Pharmingen, USA) and apoptotic rate was calculated by flowjo software as described previously.^56^

#### Wound healing assays

Would healing assays were performed to measure the migratory ability of HCC cells. The transfected cells were cultured in the 6-well plates. When the density of HCC cells in the 6-well plate reached more than 90% confluence, a 10 μl pipette tip was used to scratch the cells inside the six-well plate to produce a thick and uniform track. Floating dead cells were washed away with PBS and replaced with a serum poor basal medium for continued culture. The scratch was imaged and analyzed at 0 h, 10 h, or 20 h. Wound healing was monitored on a light microscope.

#### Transwell migration and invasion assays

Transfected or drug-treated HCC cells were analyzed using Transwell chambers with Matrigel (Costar, Corning Inc., NY, USA). The same cell number was taken from control and treatment groups, resuspended with serum-free basal medium, and placed in the upper chamber culture. The lower chamber was filled with a complete medium containing 10% serum as a chemoattractant. The chambers were removed after 24 h of incubation and processed for crystal violet staining for 30 min. The migrated and invaded cells were counted and imaged by microscopy after air drying as described previously.^57^

#### Total RNA extraction and real-time RT-PCR

Total RNA of transfected HCC cells was extracted using a rapid cell/tissue total RNA Extraction Kit (Norevizan, Nanjing, China). Reverse transcription of RNA to yield cDNA was performed using the reverse transcription Kit (Norevizan). Real-time PCR reactions were performed on a Roche 9600. Primers were provided by Shanghai Sangon biological company. Primer sequences were as follows: Nosip, forward primer 5‘-CCC AGA ACA TTC GAC TGA GCC-3’, reverse primer 5‘-TGA CAA CAG GAT CGT GGC AAG -3’. GAPDH was used as an internal reference. GAPDH, forward primer 5‘-CAG CCT CAA GAT CAT CAG CA-3’, reverse primer 5‘-TGT GGT CAT GAG TCC TTC CA -3’. PCR parameters included: initial denaturation at 95°C for 10 min, 40 cycles of 95°C for 5 sec; 63°C for 30 sec and 72°C for 30 sec, and final extension at 72°C for 5 min. Relative gene expression was measured using the 2^ΔΔCq^ method.

#### Western blotting analyses

HCC cells after transfection or drug treatment for 48 h were digested from 6-well plates using pancreatin and transferred into 1.5 ml EP tubes, followed by addition of protease inhibitors (Beyotime, China) to RIPA (Beyotime, China) at a ratio of 100: 1. Cells in EP tubes were lysed using the configuration completed RIPA lysis solution at low temperature to obtain proteins. Protein concentrations in HCC cells were determined using BCA (Beyotime, China), and loading buffer was added to the proteins after quantification and placed in a metal bath at 100°C for 5 min to denature the proteins to obtain loading proteins. The loading proteins were separated using polyacrylamide gel electrophoresis. The proteins on the separating gel were transferred to a polyvinylidene difluoride (PVDF) membrane, and the PVDF membrane with proteins were put into 5% non-fat milk for blocking treatment. The blocked membrane strips were placed in different antibodies overnight at 4°C with refrigerator shaking. The following day, incubation of the corresponding secondary antibodies was continued for 2 h at room temperature after the primary antibodies were washed away using TBST. Finally, Western blot reactions were performed as described previously.^58^

#### Detection of NO formation by DAF-2DA

To investigate the relationship between NOSIP and NO synthesis, we transfected NOSIP small interference and overexpression plasmids in Hep3B cells and SNU-449 cells for 48 h, respectively. After discarding the old medium and adding DAF-2DA working solution at a concentration of 25 μM, the cells were co-cultured for 45 min in dark environment and then washed triple with PBS buffer to adequately remove the DAF-2DA that had not entered the cells. Then the cells were photographed by fluorescence microscopy to observe the fluorescence intensity of intracellular NO.

### Quantification and statistical analysis

T-tests were used for two-group comparisons, and ANOVA was performed for multiple group comparisons. GraphPad Prism 8.0 was used for plotting and statistical analysis. *P* < 0.05 was considered statistically significant.

## Data Availability

• This paper analyses existing, publicly available data. These accession numbers for the datasets are listed in the key resources table. All data reported in this paper will be shared by the lead contact upon request. • This study did not report original code. • Any additional information required to reanalyze the data reported in this paper is available from the lead contact upon request.
